# Neonatal leptin treatment reverses the bone-suppressive effects of maternal undernutrition in adult rat offspring

**DOI:** 10.1038/s41598-017-07500-5

**Published:** 2017-08-09

**Authors:** Elwyn C. Firth, Greg D. Gamble, Jillian Cornish, Mark H. Vickers

**Affiliations:** 1 Liggins Institute, University of Auckland, Auckland, New Zealand; 2 Department of Exercise Sciences, Faculty of Science, University of Auckland, Auckland, New Zealand; 3 Bone and Joint Research Group, Department of Medicine, University of Auckland, Auckland, New Zealand

## Abstract

Alterations in the early life environment, including maternal undernutrition (UN) during pregnancy, can lead to increased risk of metabolic and cardiovascular disorders in offspring. Leptin treatment of neonates born to UN rats reverses the programmed metabolic phenotype, but the possible benefits of this treatment on bone tissue have not been defined. We describe for the first time the effects of neonatal leptin treatment on bone in adult offspring following maternal UN. Offspring from either UN or *ad libitum*-fed (AD) rats were treated with either saline or leptin (2.5 µg/ g.d on postnatal days (D)3–13) and were fed either a chow or high fat (HF) diet from weaning until study completion at D170. Analysis of micro-tomographic data of the left femur showed highly significant effects of UN on cortical and trabecular bone tissue indices, contributing to inferior microstructure and bone strength, almost all of which were reversed by early leptin life treatment. The HF fat diet negatively affected trabecular bone tissue, but the effects of only trabecular separation and number were reversed by leptin treatment. The negative effects of maternal UN on skeletal health in adult offspring might be prevented or attenuated by various interventions including leptin. Establishment of a minimal efficacious leptin dose warrants further study.

## Introduction

Developmental Programming refers to the process whereby a stimulus experienced at a particular developmental stage results in alteration in phenotype (structure, function or behaviour) that is retained in later life. Such alterations can alter risk for a range of non-communicable diseases including type 2 diabetes and cardiovascular disease. Even subtle environmental stimuli have been shown to produce lasting changes in phenotype^1^, but must be delivered during critical periods of developmental plasticity, which extend from pre-conception through gestation to infancy before waning across the remainder of the life course.

The most extensively characterised environmental influence known to alter phenotype of human and animal offspring is via alterations in maternal nutrition at various stages during pregnancy and early life. Undernutrition (UN) in humans and animals increases risk of (pre-) disease states including insulin resistance, type 2 diabetes, dyslipidaemia, obesity, reduced physical activity, altered appetite regulation, hypertension, cardiovascular disease, and sarcopenia^2^. A postnatal obesogenic diet markedly exacerbates some programmed phenotypes^3^.

Given that maternal UN gives rise to obesity and leptin resistance in later life, considerable attention has centred on the role of the adipokine leptin in the development of an aberrant offspring phenotype. In a range of experimental models neonatal leptin treatment reversed the postnatal consequences associated with both leptin deficiency and maternal UN^4–10^. Complete reversal of this UN phenotype in later life, including normalization of obesity, blood pressure and insulin sensitivity has been demonstrated^6^ following neonatal leptin treatment; the effects of leptin were specific to UN offspring with no significant effects of treatment observed in offspring of control pregnancies. Importantly these effects have been replicated in other model species^11^.

In the context of developmental programming via maternal UN and leptin as an intervention strategy, little attention has been paid to a potential role in bone development. Recent evidence has linked low birthweight and poor adult bone health^12^ with the effects of growth restriction on bone health and increased fracture risk exacerbated in the setting of rapid postnatal weight gain^13^. Leptin itself is essential for normal bone growth, maturation, and turnover. Recent data suggest that leptin acts peripherally to couple bone acquisition to energy availability and that limited transport across the blood brain barrier ensures that the growth promoting effects of peripheral leptin are not constrained by the hormone’s CNS-mediated anorexigenic actions^14^. In the present study we therefore investigated the role of maternal UN, neonatal leptin treatment, a post-weaning high fat (HF) diet and the interactions therein, on bone morphology in adult female rat offspring. We hypothesised that these factors would be associated with significant changes in relevant features of bone morphology in adult offspring at the age of 170 days.

## Methods

The animal model utilised has been described in detail elsewhere^6, 15^, and all methods were performed according to the guidelines and regulations of the University of Auckland Animal Ethics Committee, which approved the project.

**Figure 1:**
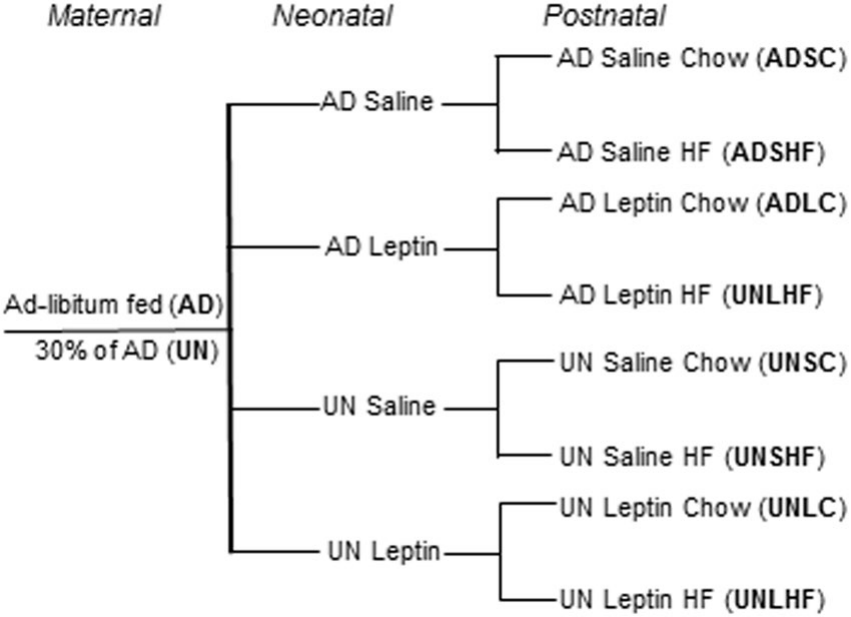
Schematic of the experimental design, in which rats (n = 8/group) were exposed to combinations of programming (UN = undernutrition vs AD = ad libitum feeding during pregnancy), Leptin vs Saline administration on neonatal days 3–13, and High Fat (HF) vs Chow (C) post-weaning diet.

Female Wistar rats (100 days of age) were time mated using an estrous cycle monitor (EC-40, Fine Science Tools, CA, USA). After confirmation of mating, females were individually housed under standard conditions (25 °C ambient temperature and 12:12 light/dark cycle in standard rat cages, with free access to water). Bodyweights and food intake were measured daily. Pregnant dams were randomly assigned to either normal *ad libitum* (AD) feeding or 30% of AD intake (UN). Pups were weighed at birth, litter size adjusted to 4 females and 4 males per litter to standardise pre-weaning nutrition, and pups from UN dams cross-fostered onto AD dams. From postnatal day 3 (D3) to D13, pups received either saline (S) or recombinant rat leptin by 2.5 µg/gBW/d subcutaneous injection (L). All dams were fed AD until weaning (D21), when offspring were weight-matched within maternal and treatment group and placed on either a standard rat chow (C) or HF diet (45% kcal as fat, D12451, Research Diets, NJ, USA). This resulted in a fully balanced 2 × 2 × 2 experimental design (maternal diet, leptin treatment and post-weaning diet as factors) with n = 8 females per group examined (Fig. 1). Male offspring were used in an independent study^5^. Whole body composition was assessed by dual-energy x-ray absorptiometry (DXA, Hologic, Waltham, MA) and the rats were killed at D170 by decapitation while anaesthetized. The significance of the effect of each of the three factors on whole body bone mineral content (BMC) and areal bone mineral density (_a_BMD) was determined.

At necropsy, the left femur was dissected from surrounding soft tissue, fixed in neutral formal saline and stored in 70% ethanol. After removal of soft tissue remnants, bone length (sliding calliper) was measured from femoral head to most distal aspect of the medial femoral condyle, and micro-computed tomography (microCT) scanning was conducted (Skyscan 1172 scanner, x-ray voltage 80 kV, 1 mm aluminum filter, isotropic voxel size 8 μm) as previously described^16^. After standardized reconstruction using NRecon software, the datasets were analyzed using CTAn software (Bruker microCT). The regions of interest were respectively from 1.4 mm proximal to the distal femoral physis and extending 2.4 mm proximally (trabecular) and 5.6 mm proximal to the physis, extending 0.8 mm proximally (cortical).

In cortical bone, measures analysed were volume enclosed by periosteal bone surface or total volume (TV), bone volume (BV), medullary volume (Md.V), mean cortical thickness, total cortical porosity (Po(tot)), mean polar moment of inertia (MMI_polar_), and mean cortical tissue mineral density (TMD). In trabecular bone, the outcome measures analysed were the tissue volume selected for analysis (TV), the volume of bone (BV) within TV (BV/TV), trabecular thickness (Tb.Th), separation (Tb.Sp), number (Tb.N), and connectivity density (Conn.Dn). For illustrative purposes a representative image was chosen by selecting the animal with the median value of trabecular separation in each of the 8 groups.

### Statistical analyses

The significance of each of programming (UN vs AD maternal nutrition during pregnancy), leptin administration (L or S treatment as neonates), and post-weaning diet (HF or C), and the interactions therein, on the named bone parameters adjusted for bodyweight (except those which already had a body-size adjustment (BV/TV, MMI_polar_)) at D170 were determined using three-way factorial analysis of variance (ANOVA), after a linear adjustment (femur length) included in a sensitivity analysis was shown to have no effect. All tests were two-tailed and P < 0.05 was considered significant. Data were analysed using SAS (v9.4, SAS Institute Inc, Cary NC). Additional analyses were performed (analysis of covariance (ANCOVA)) taking into account body weight, bone length and nesting animals within the cages they shared. Significant main and or interaction effects were further explored using the Tukey’s method. Analyses for different endpoints were prioritized and no adjustment for multiplicity between endpoints was performed.

## Results

Phenotypic differences described previously^6^ included maternal UN resulting in significantly reduced birth weights, catch-up growth and increased adiposity in later life of offspring. These effects were exacerbated in the presence of a post-weaning HF diet, and reversed in offspring treated with leptin.

### Body weight

There were obvious between-group differences at D170 in absolute body weights (Table 1). There were no differences in body weights between saline-treated AD and UN offspring and an overall effect of a HF diet on body weight (p < 0.001). There was a significant (P = 0.035) interaction between maternal diet and treatment with leptin. Post-hoc comparisons showed that leptin was significantly more effective in reducing weight in UN than AD offspring.

**Table 1:** Group mean (±SEM, n = 8 per group) whole body bone mineral content (BMC) and areal bone mineral density (_a_BMD) measured by dual energy x-ray absorptiometry (DXA), femur bone length, and body weight at D170 of 8 groups of female rats exposed to combinations of programming (UN = undernutrition vs AD = ad lib feeding during pregnancy), leptin (L) vs saline (S) administration on neonatal days 3–13, and high fat (HF) vs chow (C) diet post-weaning.

Treatment group	BMC (g)	_a_BMD (g/cm^2^)	Bone length (mm)	Body weight at D170 (g)
**ADSC**	9.93 (0.39)	0.170 (0.001)	33.8 (0.21)	341 (9.66)
**ADLC**	9.64 (0.43)	0.168 (0.002)	32.75 (0.22)	325 (12.5)
**ADSHF**	11.61 (0.52)	0.174 (0.002)	34.8 (0.28)	377 (14.9)
**ADLHF**	10.64 (0.04)	0.168 (0.001)	32.99 (0.32)	373(13.4)
**UNSC**	8.43 (0.18)	0.163 (0.001)	32.48 (0.11)	307 (6.7)
**UNLC**	8.28 (0.23)	0.164 (0.002)	32.19 (0.14)	287 (7.5)
**UNSHF**	11.48 (0.50)	0.163 (0.003)	32.73 (0.20)	413 (19.5)
**UNLHF**	9.39 (0.31)	0.173 (0.003)	33 (0.49)	328 (13.6)
**Effects**				
UN Programming	0.001	0.01	0.0009	0.007
Neonatal Leptin	0.002	—	—	0.005
Postnatal HF Diet	0.001	—	—	0.001
**Interactions**				
UN Programming*Leptin		0.009	0.00006	0.035
UN Programming* x HF diet		—	—	—
Leptin*HF diet	0.02	—	—	—
UN Programming*Leptin*HF diet	—	0.048	0.0354	—

### Bone length

There was a significant effect of UN, UN-L interaction, and UN-L-HF interaction but no significant direct effect of L or HF. Femur length of UN offspring was 0.91 mm (95% CI 0.51, 1.31) less than that of AD offspring (P < 0.0001), and was 0.65 mm (95% CI 1.04, 0.25) less in L than S groups (P = 0.0021) except in UNLHF group which was slightly longer than in UNSHF and ADLHF groups. In offspring fed the post-weaning HF diet femoral length was 0.50 mm (95% CI 0.90, 0.10) more than in those fed normal chow.

### Body composition

There were significant direct effects of UN, L, and HF diet (Table 1) on BMC. UN was clearly associated with a lower BMC in three of the groups (SC, LC, and LHF) with the clear exception of the UNSHF group which had BMC similar value to that of ADSHF. BMC was strongly correlated with body weight (r^2^ = 0.85, p < 0.001). The L groups had consistently lower values than their S control counterparts. All HF groups had obviously higher BMC than C groups. There was a significant direct effect of UN on _a_BMD (Table 1), and significant UN-L and UN-L-HF interactive effects.

### Micro-computed tomography

**Figure 2:**
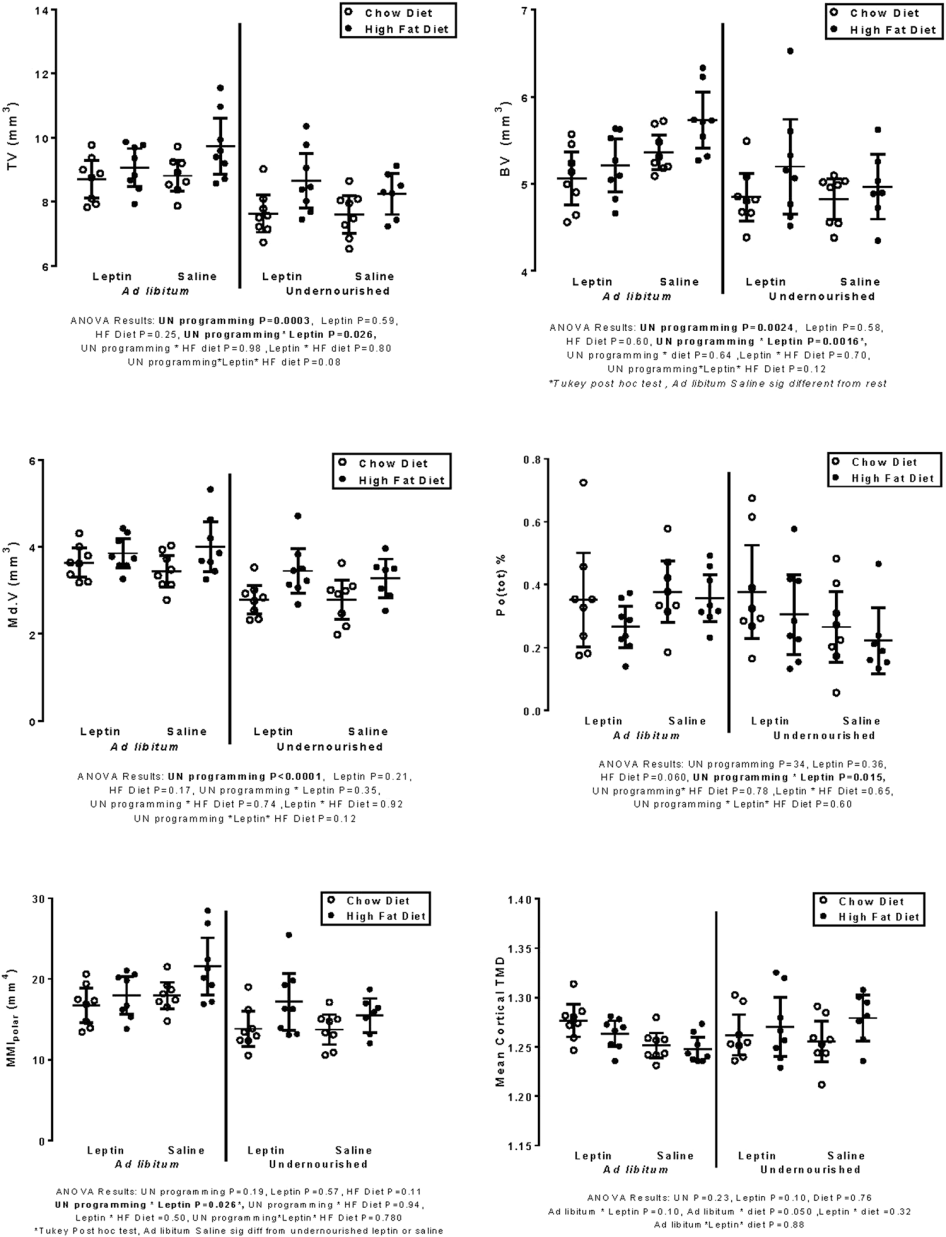
Cortical bone. Descriptive data (mean, 95% CIs) and ANCOVA results of direct and interaction effects of combinations of programming (UN = undernutrition vs AD = ad libitum feeding during pregnancy), Leptin vs Saline administration on neonatal days 3–13, and High Fat (•) vs Chow (◦) diet post-weaning on six bone morphometry measures. TV = total volume, BV = Bone volume, Md.V = Medullary volume, Po(tot) = % total porosity, MMI_polar_ = polar moment of inertia, and TMD = mean cortical tissue mineral density. Statistically significant (P < 0.05) results are in bold.

**Figure 3:**
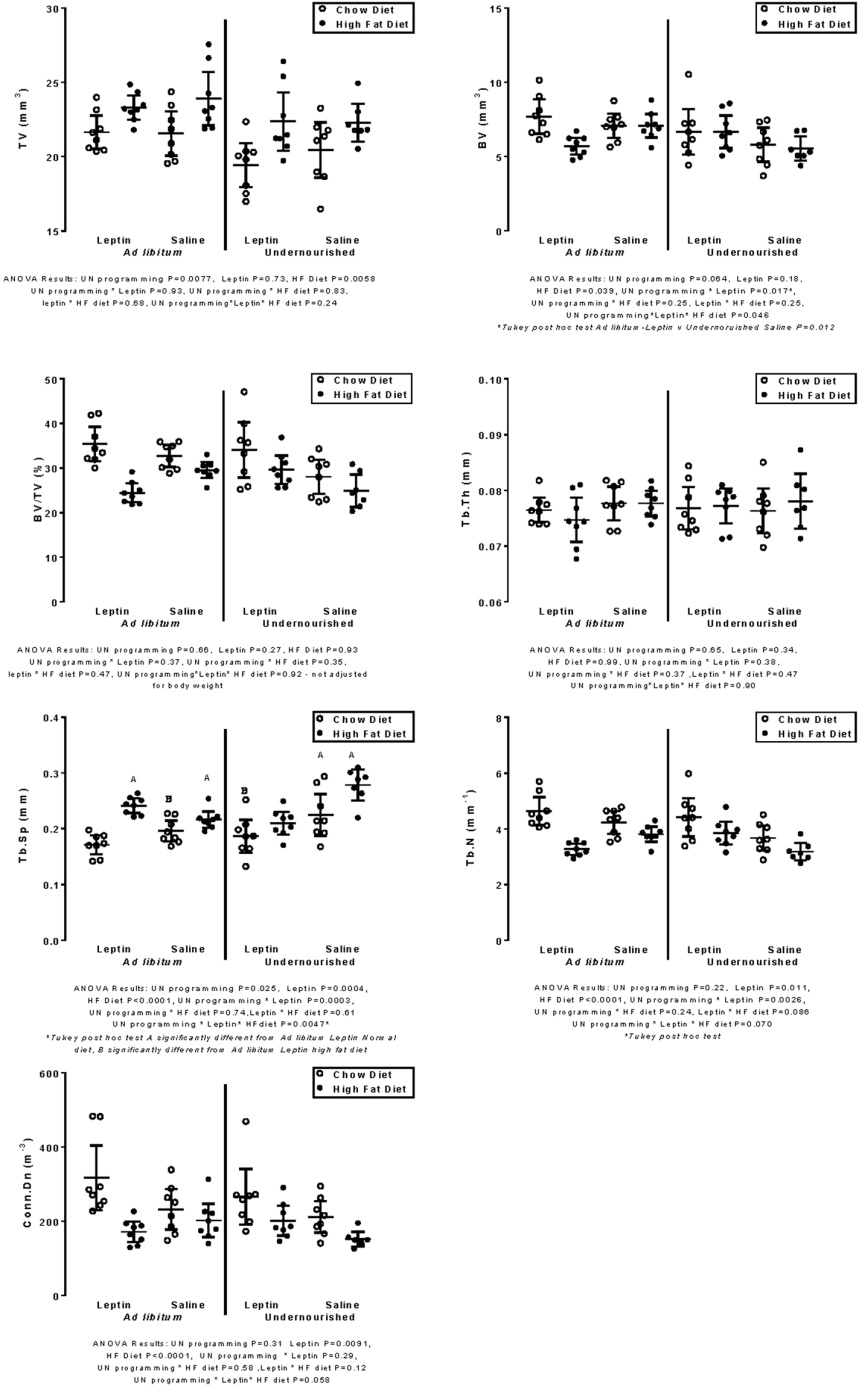
Trabecular bone. Descriptive data (mean, 95% CIs) and ANCOVA results of direct and interaction effects of combinations of programming (UN = undernutrition vs AD = ad libitum feeding during pregnancy), Leptin vs Saline administration on neonatal days 3–13, and high fat (•) vs chow (◦) diet post-weaning on seven trabecular bone morphometry measures. TV = total volume, BV = Bone volume, Tb.Th = trabecular thickness, Tb.Sp = trabecular separation, Tb.N = trabecular number, Conn.Dn = Connectivity density. Statistically significant (P < 0.05) results are in bold.

The means and 95% CI of chosen dependant variables are shown in Figs 2 and 3, which also show the significance of the main effects, interaction effects, and Tukey post hoc tests. Data of cortical thickness and tissue mineral density are shown for completeness despite having neither direct or interaction effects and significant between-group differences in least square means.

### Cortical bone

The mean TV of offspring of UN mothers was 8.09 mm^3^ (95% CI 7.82, 8.377), 0.90 mm^3^ less (P < 0.0001) than that of offspring of AD mothers (8.72, 9.27).

The BV in offspring of UN mothers was 4.99 mm^3^ (4.85, 5.13), 0.32 mm^3^ smaller (P = 0.0024) than that of AD offspring (5.17, 5.45 mm^3^).

Cortical Md.V in UN offspring was 3.11 (2.93, 3.29), 0.58 mm^3^ smaller (P < 0.0001) than in AD offspring (3.51, 3.87 mm^3^).

The MMI_polar_ in UN offspring was 15.31 mm^4^ (14.25, 16.38), 2.94 mm^4^ less (P = 0.0003) than in offspring of AD mothers (17.20, 19.29).

Cortical thickness, tissue mineral density, and Po(tot) were not significantly influenced by any of the three main effects. The only significant interaction effect was that of UN-L, which was evident in cortical BV, Po(tot), and MMI_polar_ (Fig. 2).

### Trabecular bone

The mean TV of UN offspring was 19.86 (20.52, 21.85) 1.33 mm^3^ smaller (P = 0.0077) than in AD offspring (21.85, 23.17). In offspring fed the HF diet, TV was 22.74 mm^3^ (21.96, 23.52), 1.78 mm^3^ greater (P = 0.0058) than in offspring fed the control chow diet (20.20, 21.72). The BV was 6.08 mm^3^ (5.55, 6.61) in HF-fed offspring, 0.89 mm^3^ less (P = 0.039) than in chow-fed (6.45, 7.47) offspring. The BV/TV in UN and AD groups was similar (P = 0.37), but higher in chow- than HF-fed groups (P = 0.0001), and higher in leptin- than saline-treated UN groups (P = 0.035) but lower in leptin-treated AD group fed HF diet (UN-L interaction P = 0.0167). The pattern of values was very similar to that for BV (Fig. 3).

**Figure 4:**
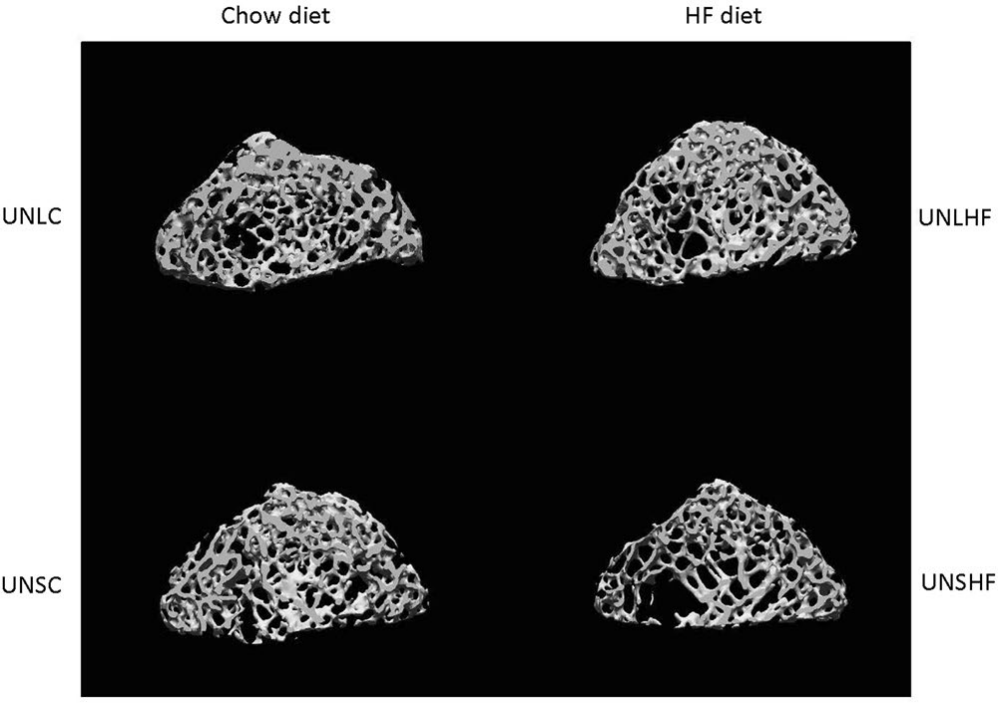
MicroCT processed images of the metaphyseal volume of interest of the animal closest to the median Tb.Sp value for the four UN groups. Abbreviations are those in text and the Fig. 1 legend. The Tb.Sp and Tb.N values (Fig. 3) are visibly most abnormal in the UNSHF image (bottom right), and reversed by leptin in the UNLHF group, values in which are similar to those of the UN chow-fed control groups.

There was no influence of any of the three main effects on Tb.Th. The Tb.Sp was significantly influenced by UN, L and HF diet. In UN offspring Tb.Sp was 0.224 mm (0.214, 0.234), 0.017 mm wider (P = 0.025) than in AD offspring (0.197, 0.217). In leptin-treated offspring Tb.Sp was 0.201 mm (0.191, 0.211), 0.029 mm narrower than in saline controls (0.221, 0.241, P = 0.0004). The differences between the four UN groups are shown in Fig. 4. Leptin reduced Tb.Sp in the UN groups, but not in AD offspring fed HF post-weaning diet, indicated by the significant interactions involving leptin and the significant post hoc Tukey tests (Fig. 3). In offspring fed the HF diet, Tb.Sp was 0.239 mm (0.227, 0.251), 0.047 mm wider (P < 0.0001) than in those fed the control chow diet (0.18, 0.203).

The Tb.N in HF groups was 3.47 (3.25, 3.69), 0.83 less (P < 0.0001) than chow-fed controls (4.08, 4.51), (P = 0.0001). The Tb.N in leptin-treated offspring was 4.07 (3.88, 4.25), 0.37 more (P = 0.011) than in saline-treated controls (3.50, 3.89), (P = 0.011), except in AD-HF offspring as shown in the significant interaction of maternal nutrition and leptin (Fig. 3).

The Conn.Dn measure of leptin-treated offspring was 242.7 (219.1, 266.3), 47.4 mm^−3^ higher (P = 0.009) than in saline controls (171.1, 219.5). In chow-fed offspring, Conn.Dn was 265.0 (238.2, 291.8), 92.01 higher (P < 0.0001) than in the HF-fed offspring (145.3, 200.7).

The UN-L interaction on Tb.Sp, Tb.N, BV and BV/TV (Fig. 3), was the only significant interaction in trabecular bone.

### Interactions

To clarify the between-group interactive effects the data were re-analysed after adjustment for post-weaning diet, to show the significance estimates of the effect of maternal nutrition and of postnatal leptin (post hoc Tukey testing).

### Cortical

In cortical TV and BV the UN-L interaction was significant (P = 0.026 and P = 0.002 respectively) and adjusted mean values in Saline-treated were higher than in Leptin-treated AD offspring but were higher in Leptin than saline-treated UN offspring. Post-hoc adjusted mean TV was significantly lower in UN-S than in AD-S, AD-L or UN-L (P < .0001, 0.003, 0.052 respectively); BV of the UN-S offspring group was significantly lower (P < 0.0001) than only that of the AD-S group.

The UN-L interaction for cortical Po(tot) and MMI_polar_ (P = 0.015 and P = 0.005) and the association pattern was similar, with lower porosity values in the AD-L than the AD-S group, and lower values in the UN-S than the UN-L group; Tukey post hoc estimates were non-significant for Po(tot), but were for MMI_polar_ which was significantly lower in UN-S than the AD-L and AD-S groups (P = 0.003 and P < 0.0001 respectively) but not different from the UN-L offspring group (P = 0.056).

### Trabecular

The UN-L interaction was for BV (P = 0.017), and the post hoc test suggested that maternal UN-L offspring had the same BV as the AD-S or AD-L groups, while the real difference (P = 0.0117) was between adjusted means of the UN-S and AD-S offspring groups.

For Tb.Sp the UN-L interaction (P = 0.0003) suggested that UN-L offspring were not different to offspring of AD mothers given Leptin or Saline, in all of which Tb.Sp was less than that in the UN-S offspring group (P < 0.0001–0.0002). The same effect was shown in Tb.N, which was less in the UN-S group than in UN-L, and in AD-S or AD-L groups (P = 0.002–0.03), the UN programming effect having been reversed by leptin. For Conn.Den the interaction was not significant, but the post hoc test indicated that the value for the UN-S group was significantly lower than that of the AD-L group (P = 0.035; P = 0.06 for UN-S vs UN-L group).

Comparing the P values of the 7 effects (3 main and 4 interactive) on the 6 cortical measures before and after adjustment for bodyweight at D170, 6 of the 42 pairs were different; in 5 of the 6 the value after adjustment was non-significant, and in one (the UN-L interaction P value for TV) had become significant.

Of the 49 P value pairs of the 7 trabecular measures, the significance in 44 was not different after adjustment. In 2 of the 5 cases (UN programming effect on BV, and leptin-HF diet effect on BV/TV ratio) the effect became non-significant after adjustment, and the effect of both HF diet and the UN-L_HF interaction on BV, and of leptin on BV/TV became significant.

## Discussion

Alterations in the early life environment can result in increased risk of various metabolic and cardiovascular disorders in later life, which are amplified in the setting of a postnatal obesogenic environment. Such effects of early life “developmental programming” on bone tissue development after maternal undernutrition have not been well defined. The UN offspring treated with leptin as neonates showed a reversal of the programmed metabolic phenotype including normalisation of body weight, fat mass and leptin concentrations in adulthood^6^. The present study therefore examined the effects of maternal UN, neonatal leptin treatment, and post-weaning HF obesogenic diet on adult bone measures.

Leptin is an important osteogenic factor in early life^17^, prevented tail suspension-induced bone loss changes in cortical and trabecular bone^18^, exerted an effect on both bone cells and physeal chondrocytes^19^ and a dose-dependent effect on rat femur length, and reversed the caloric restriction-related reduction in tibia length in young mice^20, 21^. Bone length was overall significantly shorter in UN compared to control offspring in the present study, and the UN-L interaction indicated that neonatal leptin reduced bone length in AD offspring groups fed a post-weaning HF diet, and had the opposite effect in the UN group fed the HF diet.

Regarding cortical bone measures, only UN programming had a significant direct effect. The TV, BV, Md.V, and MMI_polar_ were less in UN than AD groups, and the descriptive figures overlay each other very closely. The effect on MMI_polar_ was because a greater proportion of BV in UN than AD was disposed closer to the diaphyseal centroid; Po.(tot) of cortical bone may have had an effect but with individual values of only ~0.1–0.4% of bone volume this seems unlikely. Although diaphyseal bone size and cortical apparent bone mineral density are reciprocally related in active people^22^ and animals^23^, the differences in bone size were small, and the other effects of UN including lower physical activity and elevated HF intake in the UN rats may have played a role in the negative effect on MMI_polar_ differences.

In cortical bone the only significant interaction was that of leptin and UN programming in cortical BV, Po(tot), and MMI_polar_. In UN-HF groups the effects of the UN were positively affected by neonatal leptin administration, with an opposite effect in the AD groups. The same was evident after adjustment, the real differences being between the UN-S and the other groups, indicating the mitigation of the effects of maternal UN by leptin. This leptin effect was more evident in cortical TV than BV, the difference between UN-S and UN-L group means proportionally greater in TV than in BV. The phenotypic effect of programming was that volume fraction of cortical BV to that of bone size (diaphyseal tissue volume) was highest in the UN-S group, with the bone itself small but maintaining cortical bone volume, possibly through lower bone turnover, since the porosity was lowest in UN-S groups.

In trabecular bone, microstructure is largely assessed by analysing size (thickness), number, and connections between trabeculae, and the distance between them. Only trabecular TV and Tb.Sp were significantly influenced by the direct effect of UN programming, underscoring that the effects of dietary restriction during pregnancy can result in less robust development (smaller TV and greater Tb.Sp, apparently “for life”), since these features were demonstrated in adult rat offspring. Lack of compensatory bone development has been long recognised^24^ and that it was rescued by neonatal leptin treatment strongly indicates leptin is an important mediator of bone metabolic and morphological programming. The Tb.Th was not significantly influenced by any effect, concurring with findings of others^25, 26^, perhaps consistent with Tb.Th having the least adaptive tendency of trabecular parameters measured in animals with bodyweights differing by six orders of magnitude^27^ because of the imperative of controlling Tb.Th to maintain mechanical homeostasis and vascular-mediated remodelling^28^.

In four trabecular measures, BV, BV/TV, Tb.Sp and Tb.N, there were significant UN-L interactions. The UN-L group had adjusted mean values significantly different from those of the AD-L, AD-S and UN-L groups (the latter three had closely similar values). Leptin treatment largely removed the UN programming-induced inferior structural bone quality of the UNSHF group, but had no effect in the two AD groups.

The HF diet, in contrast to its having no significant direct effect on cortical bone tissue, had a highly significant effect on five trabecular bone morphometry measures (Fig. 3). In only TV was the effect positive in terms of bone properties that might contribute to bone strength, with TV of HF groups being 8.4% greater than that of chow-fed groups. The amount and structural arrangement of bone tissue in HF-fed groups was apparently inferior to that of the chow-fed groups, in that trabecular BV, Tb.N and Conn.Dn were respectively 14.6%, 22.6%, and 53% higher in Chow- than HF-fed groups, and the inter-trabecular distance (Tb.Sp) was 24.5% greater in HF- than in Chow-fed groups. The significant direct effect on trabecular BV by HF diet is in agreement with recent findings in rat femur^25^ and mouse tibia^26^. The Tb.Sp in HF groups was higher than in chow diet control groups, and was highest in the UNSHF group, which leptin (UNLHF) normalised to values of the UN chow-fed groups. There was no significant interactive effect of HF diet with either programming or leptin on trabecular outcome measures, except as part of the UN-L-HF interaction in trabecular BV and Tb.Sp (P = 0.046 and 0.0047 respectively).

The morphology measures were weight-adjusted because of the possible confounding effect of bodyweight, which was starkly different between groups, due to a highly significant increase in fat mass by the HF diet (P < 0.0001)^6^. Increased body weight positively influenced bone mass, and analysis of the unadjusted values had revealed significant effects of HF diet on several femoral cortical outcome measures. However, the various influences on bone tissue cannot be determined from such data because the positive effects of bodyweight on bone mass, due to the mechanical effects of supporting and moving a larger body weight, cannot be separated from those of the obesity-associated physical and metabolic phenotype induced by the programming and post-weaning diet interventions. Obese phenotype induced by HF diet in rodents has previously been shown to have negative effects on bone tissue^25, 26, 29^.

In contrast to the significant UN-L interaction which attenuated the programming effect in both cortical and trabecular bone measures, HF diet had no significant interaction with leptin in either tissue. This indicates that leptin influenced the negative effects of diet only through the programming-leptin-diet interaction in trabecular BV, Tb.N and Tb.Sp. At a microstructural level the effects of HF diet thus appear to be summative with those of UN programing, but only the latter’s effects were significantly (for Tb.Sp and Tb.N) blunted by leptin. This resulted in higher BV in Leptin- than Saline-treated counterpart groups with the exception of the ADLHF group, where leptin treatment decreased adult BV, opposite to the effect in the UNLHF group in which leptin treatment corrected the low BV value of UNSHF to control group levels, as shown above for Po(tot) in cortical bone. These significant effects would appear to be due to the UN-L and the UN-L-HF interactions, implying that programming effects associated with nutrition during pregnancy were modulated by neonatal exogenous leptin. The peripheral effect of leptin has been shown in mice to be due to increased osteoblast numbers, bone formation and bone growth rate^30^.

The most vulnerable offspring group (UNSHF) had been exposed to the effects of both UN during pregnancy and a post-weaning HF diet and had negative outcomes (adiposity, fat mass, sedentary activity, elevated fasting plasma biomarker concentrations^6^). The metabolic phenotypic effects, measured 23 weeks after neonatal leptin administration ceased, were reversed in the UNLHF group^6^. The most abnormal bone outcome measures (cortical Po(tot) and trabecular BV, Tb.Sp, Tb.N, and Conn.Dn) also were in the UNSHF group, and were attenuated by leptin to normal values of the control groups. But some indices did not contribute to bone strength or quality, because of no significant direct or interaction effect (Tb.Th) or only very small between-group differences (Po(tot), TMD). That bone tissue differences were present 23 weeks after leptin administration emphasises that early life programming needs to be considered in interpreting results of studies on the varied^31^ effects of leptin on bone, and in choices of experimental design for bone studies in rodents.

The demonstrated effects on bone micromorphology measures did not appear to be a secondary response of the femur to bodyweight at D170, since higher group bodyweight was not associated with the highest femoral BV. Femur length as a linear adjustment for body size had had no effect in a sensitivity analysis. Thus the relationship between the unadjusted and bodyweight-adjusted probability values (reflecting the effect of bodyweight on whole body phenotype and on bone cells/tissue respectively) were largely the same in both heavier and lighter groups. Even more striking was that adjustment for femoral trabecular micromorphology measures (Tb.Sp, Tb.N, Conn.Den and Tb.Th) for the effect of bodyweight did not alter the pattern of significance values, at all. The interpretation was that there was a residual effect of UN programming and of leptin independent of the effects of bodyweight. Extra bodyweight, largely associated with HF diet, with or without the programming effect, was not itself the factor responsible for the effects associated with the HF diet. This may be because of other environmental factors associated with HF diet, including particular dietary components, the induced pro-inflammatory state, or the lack of physical activity (and thus of muscle forces acting on the skeleton) in the pair-caged environment.

The UNSHF group was markedly hyperleptinemic compared to other groups^6^ and the mean plasma leptin concentration in these UNSHF offspring was striking in being 2–8 times higher than the other groups in this study (or any other encountered in the literature consulted). This may reflect a number of factors. Maternal UN followed by a postnatal obesogenic diet creates a widely discrepant nutritional mismatch following the “predictive adaptive response” concept^32^ whereby a fetus exposed to maternal UN adapts to an expected post-natal environment of scarcity, but experiences a contrasting obesogenic postnatal environment. This leads to a marked exacerbation of programming effects, and possibly the marked hyperleptinemia observed in this and other studies.

The dose of leptin in the present study was in line with that used in other rat studies^33, 34^ and utilised a homologous system (recombinant rat leptin), whereas some have utilised a non-homologous system with human leptin treatment, and this difference may elicit some differential effects as related to diuretic naturesis^35^. This and other differences between study protocols have hampered understanding of leptin’s role in bone biology. The exact reasons underlying our observations above thus remain undefined, but most likely are linked to a recent indication^36^ that growth restricted and leptin-replete neonates respond differently to leptin administration, conferring respectively protective effects and leptin resistance-related effects such as resistance to the anorectic effect of leptin, associated with higher food intake, bodyweight gain, and retroperitoneal fat mass^37^, and lower thyroid hormone concentrations^38^.

Recent data suggest that bone metabolism is more sensitive to leptin than the levels required to alter regulation of pathways directly related to energy metabolism, at least in mice^14^. The marked hyperleptinemia in our UNSHF group likely indicates that the degree of leptin resistance in chondro-osseous tissues was profound, but was nevertheless rescued from the UN programming effects by exogenous leptin in early life. Future work could focus on determining the minimal exogenous dosage regimen, to determine if and how the risk of disadvantageous effects in replete neonates might be avoided.

We conclude that maternal UN had negative effects on offspring cortical and trabecular bone morphology, which persisted into adulthood but were reversed by leptin administration early in the neonatal period. Programming effects on metabolism were amplified in rodents by consumption of a HF diet, which had negative effects on trabecular bone tissue which were little attenuated by the leptin treatment. The lowest effective dosage for reversal of programming requires further study. If the risk of negative effects of leptin therapy on metabolism can be averted, leptin might become part of early-life preventive strategies aimed at reversing the now expected detrimental, life-long programming effects on bone associated with poor maternal nutrition.
